# The epidemiology of NCAA men’s lacrosse injuries, 2009/10-2014/15 academic years

**DOI:** 10.1186/s40621-017-0104-0

**Published:** 2017-03-06

**Authors:** Zachary Y. Kerr, Adam Quigley, Susan W. Yeargin, Andrew E. Lincoln, James Mensch, Shane V. Caswell, Thomas P. Dompier

**Affiliations:** 1 Department of Exercise and Sport Science, University of North Carolina, 313 Woollen Gym CB#8700, Chapel Hill, NC 27599-8700 USA; 2 Department of Exercise Science, University of South Carolina, Blatt Physical Education Center, Columbia, SC 29208 USA; 3 Department of Exercise Science, University of South Carolina, PHRC 226, Columbia, SC 29208 USA; 4 MedStar Sports Medicine Research Center, 201 E. University Parkway, 764 Bauernschmidt Bldg, Baltimore, MD 21218 USA; 5 George Mason University, Sports Medicine Assessment, Research & Testing (SMART) Laboratory, 10900 University Blvd. MS 4E5, Manassas, VA 20110 USA; 6 Datalys Center for Sports Injury Research and Prevention, 401 W. Michigan St., Suite 500, Indianapolis, IN 46202 USA

**Keywords:** College sports, Injury rates, Lacrosse, Checking, Prevention

## Abstract

**Background:**

Participation in lacrosse has grown at the collegiate levels. However, little research has examined the epidemiology of collegiate men’s lacrosse injuries. This study describes the epidemiology of injuries in National Collegiate Athletic Association (NCAA) men’s lacrosse during the 2009/10–2014/15 academic years.

**Methods:**

Twenty-five men’s lacrosse programs provided 63 team-seasons of data for the NCAA Injury Surveillance Program (NCAA-ISP) during the 2009/10–2014/15 academic years. Injuries occurred from participation in an NCAA-sanctioned practice or competition, and required attention from an AT or physician. Injuries were further classified as time loss (TL) injuries if the injury restricted participation for at least 24 h. Injuries were reported through electronic medical record application used by the team medical staff throughout the academic year. Injury rates per 1000 athlete-exposures (AE), injury rate ratios (RR), 95% confidence intervals (CI), and injury proportions were reported.

**Results:**

Overall, 1055 men’s lacrosse injuries were reported, leading to an injury rate of 5.29/1000AE; 95%CI: 4.98–5.61. The TL injury rate was 2.74/1000AE (95%CI: 2.51–2.96). The overall injury rate was higher in competition than practice (12.35 vs. 3.90/1000AE; RR = 3.16; 95%CI: 2.79–3.58). Most injuries were to the lower extremity (58.3%), particularly the ankle (14.1%) in competition and the upper leg (14.3%) in practice. Sprains and strains were the most common diagnoses in both competition (26.9 and 23.7%, respectively) and practice (20.2% and 27.4%, respectively). Most injuries in competitions and practices were due to player contact (32.8 and 17.5%, respectively) and non-contact (29.6 and 40.0%, respectively).

**Conclusions:**

Our estimated injury rates are lower than those from previous college men’s lacrosse research. This may be due to increased injury awareness, advances in injury prevention exercise programs, or rule changes. Still, injury prevention can aim to continue reducing the incidence and severity of injury, particularly those sustained in competitions and to the lower extremity.

## Background

Participation in lacrosse has grown in popularity at both the youth and collegiate levels (National Collegiate Athletic Association 2015; US Lacrosse 2016b). There has been a constant increase in National Collegiate Athletic Association (NCAA) varsity men’s lacrosse programs over the past decade, from 211 schools and 7103 student-athletes (average squad size of 34) during the 2003/04 academic year to 350 schools and 13,165 student-athletes (average squad of 38) in the 2014/15 academic year (National Collegiate Athletic Association 2015).

Earlier literature on the epidemiology of men’s collegiate lacrosse injuries may not reflect the current game and recent changes in injury awareness, rules, protective equipment, and sport safety. The increases in participation and the number of programs may potentially come with a disproportionate amount of experienced coaching, officiating, and skill development. One college level study (Dick et al. 2007b) utilized data from the 1988/89–2003/04 academic years. At the same time, past research examining NCAA men’s lacrosse used an injury definition that considered only time loss (TL) injuries, or those injuries resulting in participation restriction time of at least 24 h (Dick et al. 2007a). Since 2009, the NCAA Injury Surveillance Program (NCAA-ISP) has captured non-time loss injuries (NTL), or injuries that resulted in participation restriction less than 24 h. Examining more recent data that include NTL injuries will characterize a larger breadth of the types of injuries occurring in men’s lacrosse. Therefore, the purpose of this study was to describe the epidemiology of NCAA men’s lacrosse TL and NTL injuries during the 2009/10–2014/15 academic years. In particular, we examine the rates and patterns of injuries to identify those that may be substantial threats to player safety and require additional medical preparedness or safety considerations.

## Methods

The NCAA-ISP uses a convenience sample of NCAA varsity sport programs from all three divisions across the United States. During the 2009/10–2014/15 academic years, 25 men’s lacrosse teams provided 63 team-seasons of data (Table 1). The 63 team-seasons represented 3.4% of all team-seasons in the NCAA during the study period. Participation varied by division and academic year. Despite this small sample, it is the largest recent sample to our knowledge of collegiate men’s lacrosse programs. The number of academic years in which each team participated varied, with an average of 3 years (range of one to six). The methodology of the NCAA-ISP is managed by the Datalys Center and has been previously described (Kerr et al. 2014) and is summarized below. This study was deemed exempt by the Research Review Board of the NCAA.

**Table 1:** Participation rates for men’s lacrosse in NCAA Injury Surveillance Program, by division and academic year

Academic year	Division I		Division II		Division III		Total	
	# participating schools	% of all sponsoring schools	# participating schools	% of all sponsoring schools	# participating schools	% of all sponsoring schools	# participating schools	% of all sponsoring schools
2009/10	5	8.6%	1	2.6%	5	3.0%	11	4.2%
2010/11	3	5.0%	1	2.4%	8	4.5%	12	4.3%
2011/12	4	6.6%	1	2.2%	7	3.7%	12	4.1%
2012/13	7	11.3%	0	0.0%	3	1.4%	10	3.1%
2013/14	3	4.5%	0	0.0%	5	2.3%	8	2.4%
2014/15	4	5.9%	0	0.0%	6	2.7%	10	2.9%
Total	26	6.9%	3	1.0%	34	2.9%	63	3.4%

### Data collection

Lacrosse programs’ athletic trainers (ATs) reported injury and exposure data in real-time through their electronic medical record application used by the team medical staff throughout the academic year. This approach allowed ATs to document injuries as part of their normal clinical practice, thus eliminating the need to enter data more than once. When an injury occurred, the AT completed a detailed report that included body part injured, diagnosis, injury mechanism, participation restriction time, and the event type in which the injury occurred (i.e., competition or practice). As ATs continued to manage injuries, they were able to view and update previously submitted information as needed. Additionally, ATs provided the number of student-athletes participating in each practice and competition.

Upon being submitted by ATs, the exported data passed through an automated verification process that conducted a series of range and consistency checks. During this export process, data related to any identifiers and personally identifiable information (e.g., name, date of birth, insurance information) were removed (Kerr et al. 2014). Data were reviewed and flagged for invalid values. The automated verification process notified the AT and data quality staff. The data quality staff would then assist the AT in resolving questionable values. Data that passed the verification process were then placed into sport-specific aggregate datasets for use by external researchers.

### Definitions

#### Injury

A reportable injury occurred as a result of participation in an NCAA-sanctioned practice or competition, and required attention from an AT or physician. We relied on the medical expertise of the team AT or physician to appropriately identify specific diagnoses.

#### Athlete-exposure

A reportable athlete-exposure (AE) was defined as one student-athlete participating in one NCAA-sanctioned practice or competition in which he was exposed to the possibility of athletic injury regardless of the duration of participation. Only varsity level practice and competition events were considered. Data from junior varsity programs, as well as any individual weight lifting and conditioning sessions, were not collected.

#### Event type

Event type was the specific event (i.e., practice, competition) in which the injury was reported to have occurred.

#### Body part injured

Body part injured was defined as the area on the body at which the student-athlete sustained his injury. Given the numerous options from which ATs could select, we grouped these values into: head/face, neck, shoulder, arm/elbow (including the upper arm and forearm), hand/wrist, trunk, hip/groin, upper leg (including thigh), knee, lower leg, ankle, foot, and other (including systemic conditions such as heat illness).

#### Diagnosis

Diagnosis was defined as the type of injury that the student-athlete sustained. Given the numerous options from which ATs could select, we grouped these values into: concussion, contusion, dislocation, fracture, inflammatory conditions, laceration, spasm, sprain, strain, stress fracture, subluxation, and other.

#### Injury mechanism

Injury mechanism was defined as the manner in which the student-athlete sustained his injury. In the NCAA-ISP, ATs selected from a pre-set list of options including: player contact (e.g., collision between two players), surface contact (e.g., abrasion from sliding on ground), equipment contact (e.g. hit by stick), contact with out-of-bounds object (e.g., running into bleachers), non-contact (e.g., injury while running), overuse, illness, infection, and other/unknown. For equipment contact, ATs could provide additional information on the specific equipment involved. We maintained injury mechanism categories with the exception of combining illness and infection, and breaking equipment contact into three distinct categories: ball contact, stick contact, and other equipment contact.

#### Participation restriction time

Injuries were categorized by the number of days of missed participation from sports (i.e., date of return to play subtracted by the date of injury). As had been done in previous research (Dalton et al. 2015; Wasserman et al. 2016; Yeargin et al. 2016), we categorized participation restriction time as NTL and TL injuries. NTL injuries were those injuries resulting in participation restriction time under 24 h. TL injuries were those injuries resulting in participation restriction time of at least 24 h. We further coded TL injuries as whether they were severe, which was defined as resulting in participation restriction time of over 3 weeks (Darrow et al. 2009). If the student-athlete or a medical professional choose to prematurely end the athlete’s season, this also met the definition. A premature end to an athlete’s season could be for medical or non-medical reasons (e.g., athlete was able to return to play but felt injury affected performance and quit).

### Statistical analysis

Injury rates and distributions of injuries from NCAA men’s lacrosse were calculated. Injury rates were calculated per 1000 athlete-exposures (AEs) overall and then specifically for competitions and for practices. Injury rate ratios (IRRs) compared injury rates between competition and practices. We also examined injury rates and distributions of injuries by body part, diagnosis, and injury mechanism. Injury proportion ratios (IPRs) compared injury distributions between competition and practices for body part, diagnosis, and injury mechanism. All IRRs and IPRs whose 95% confidence intervals (CIs) did not include 1.00 were considered statistically significant. Data were analyzed using SAS-Enterprise Guide software (version 5.2; SAS Institute Inc., Cary, NC).

## Results

### Overall frequencies and rates

During the 2009/10 through 2014/15 academic years, ATs reported 1055 men’s lacrosse injuries (Table 2). Most injuries were reported during practice (61.6%, *n* = 650). Of these injuries, 545 (51.7%) were TL injuries, with 10.9% (*n* = 115) of all injuries being severe. These 1055 injuries occurred during 199,260AE, for an injury rate of 5.29/1000AE (95%CI: 4.98–5.61). When considering only TL injuries, the injury rate was 2.74/1000AE (95%CI: 2.51–2.96). The injury rate was higher in competition than practice overall (12.35 vs. 3.90/1000AE; IRR = 3.16; 95%CI: 2.79–3.58), in the regular season (12.88 vs. 3.18/1000AE; IRR = 4.06; 95%CI: 3.48–4.72), and in the postseason (7.17 vs. 2.03/1000AE; IRR = 3.53; 95%CI: 1.97–6.30). The findings were similar when restricted to time loss injuries and severe injuries.

**Table 2:** Injury rates and 95% Confidence Intervals (CI) by time in season and type of Athlete-Exposure (AE) in NCAA Men’s Lacrosse, 2009/10–2014/15^a^

	Competition		Practice		Overall	
	# injuries in sample	Rate and 95% CI (per 1000AE)	# injuries in sample	Rate and 95% CI (per 1000AE)	# injuries in sample	Rate and 95% CI (per 1000AE)
All injuries						
Preseason	0	0.00	330	5.38 (4.80, 5.96)	330	5.38 (4.80, 5.96)
Regular season	384	12.88 (11.60, 14.17)	295	3.18 (2.81, 3.54)	679	5.54 (5.12, 5.95)
Postseason	21	7.17 (4.10, 10.24)	25	2.03 (1.24, 2.83)	46	3.02 (2.15, 3.89)
Total	405	12.35 (11.14, 13.55)	650	3.90 (3.60, 4.21)	1055	5.29 (4.98, 5.61)
All time loss injuries^b^						
Preseason	0	0.00	179	2.92 (2.49, 3.35)	179	2.92 (2.49, 3.34)
Regular season	207	6.95 (6.00, 7.89)	143	1.54 (1.29, 1.79)	350	2.85 (2.55, 3.15)
Postseason	4	1.37 (0.03, 2.70)	12	0.98 (0.42, 1.53)	16	1.05 (0.54, 1.57)
Total	211	6.43 (5.56, 7.30)	334	2.01 (1.79, 2.22)	545	2.74 (2.51, 2.96)
All severe injuries^c^						
Preseason	0	0.00	32	0.52 (0.34, 0.70)	32	0.52 (0.34, 0.70)
Regular season	47	1.58 (1.13, 2.03)	33	0.36 (0.23, 0.48)	80	0.65 (0.51, 0.80)
Postseason	1	0.34 (0.00, 1.01)	2	0.16 (0.00, 0.39)	3	0.20 (0.03, 0.42)
Total	48	1.46 (1.05, 1.88)	67	0.40 (0.31, 0.50)	115	0.58 (0.47, 0.68)

^a^Data originates from the Datalys Center for Sports Injury Research and Prevention Injury Surveillance Program, 2009/10-2014/15

^b^Includes injuries that resulted in participation restriction of at least 24 h

^c^Includes injuries that resulted in participation restriction of over 3 weeks, or the student-athlete prematurely ending his season

Among practices, the injury rate was higher in the preseason than regular season (5.38 vs. 3.18/1000AE; IRR = 1.69; 95%CI: 1.45–1.98) and postseason (5.38 vs. 2.03/1000AE; IRR = 2.65; 95%CI: 1.76–3.98). The injury rate was also higher in the regular season than postseason in practices (3.18 vs. 2.03/1000AE; IRR = 1.56; 95%CI: 1.04–2.35) and competitions (12.88 vs. 7.17/1000AE; IRR = 1.80; 95%CI: 1.16–2.79). The findings were similar when restricted to time loss injuries and severe injuries.

### Body parts injured

Most injuries occurred to the lower extremity (Table 3). In particular, commonly injured body parts included the upper leg (competition: 10.9%, *n* = 44; practice: 14.3%, *n* = 93), knee (competition: 13.3%, *n* = 54; practice: 12.3%, *n* = 80), and ankle (competition: 14.1%, *n* = 57; practice: 12.3%, *n* = 80). In addition, 10.4% (*n* = 42) and 6.5% (*n* = 42) of injuries in competitions and practices, respectively, were sustained to the head/face; a larger proportion of injuries were to the head/face in competitions than practices (IPR = 1.60; 95%CI: 1.07–2.42).

**Table 3:** Injury counts, percentages, and rates per 1000 Athlete-Exposures (AEs) by body part injured and type of event in NCAA Men’s Lacrosse, 2009/10–2014/15^a^

	Competition				Practice			
Body part	Injury count (%)	Rate and 95% CI (per 1000AE)	% NTL^b^	% Severe^c^	Injury count (%)	Rate and 95% CI (per 1000AE)	% NTL^b^	% Severe^c^
Head/face	42 (10.4)	1.28 (0.89, 1.67)	23.8	9.5	42 (6.5)	0.25 (0.18, 0.33)	19.0	14.3
Neck	5 (1.2)	0.15 (0.02, 0.29)	20.0	0.0	7 (1.1)	0.04 (0.01, 0.07)	28.6	0.0
Shoulder	40 (9.9)	1.22 (0.84, 1.60)	45.0	15.0	44 (6.8)	0.26 (0.19, 0.34)	47.7	11.4
Arm/Elbow	22 (5.4)	0.67 (0.39, 0.95)	68.2	0.0	17 (2.6)	0.10 (0.05, 0.15)	76.5	5.9
Hand/Wrist	46 (11.4)	1.40 (1.00, 1.81)	56.5	8.7	51 (7.8)	0.31 (0.22, 0.39)	43.1	19.6
Trunk	37 (9.1)	1.13 (0.76, 1.49)	67.6	0.0	74 (11.4)	0.44 (0.34, 0.55)	68.9	1.4
Hip/Groin	19 (4.7)	0.58 (0.32, 0.84)	63.2	0.0	57 (8.8)	0.34 (0.25, 0.43)	50.9	1.8
Upper leg	44 (10.9)	1.34 (0.94, 1.74)	38.6	9.1	93 (14.3)	0.56 (0.45, 0.67)	44.1	3.2
Knee	54 (13.3)	1.65 (1.21, 2.09)	29.6	35.2	80 (12.3)	0.48 (0.38, 0.59)	45.0	27.5
Lower leg	24 (5.9)	0.73 (0.44, 1.02)	54.2	20.8	55 (8.5)	0.33 (0.24, 0.42)	63.6	9.1
Ankle	57 (14.1)	1.74 (1.29, 2.19)	52.6	3.5	80 (12.3)	0.48 (0.38, 0.59)	36.3	7.5
Foot	14 (3.5)	0.43 (0.20, 0.65)	35.7	21.4	38 (5.8)	0.23 (0.16, 0.30)	52.6	13.2
Other	1 (0.2)	0.03 (0.00, 0.09)	0.0	100.0	12 (1.8)	0.07 (0.03, 0.11)	8.3	16.7
Total	405 (100.0)	12.35 (11.14, 13.55)	46.4	11.9	650 (100.0)	3.90 (3.60, 4.21)	47.4	10.3

NOTE: *NTL* non-time loss

^a^Data originates from the Datalys Center for Sports Injury Research and Prevention Injury Surveillance Program, 2009/10-2014/15

^b^Includes injuries that resulted in participation restriction under 24 h

^c^Includes injuries that resulted in participation restriction of over 3 weeks

Among all injuries, the arm/elbow had the largest proportion of NTL injuries (competition: 68.2%; practice: 76.5%), followed by the trunk (competition: 67.6%, practice: 68.9%). In contrast, the knee was the body part with the largest proportion of severe injuries (competition: 35.2%; practice: 27.5%).

### Diagnoses

A wide range of diagnoses were reported (Table 4). Common diagnoses included were sprains (competition: 26.9%, *n* = 109; practice: 23.7%, *n* = 154), contusions (competition: 24.4%, *n* = 99; practice: 12.9%, *n* = 84), and strains (competition: 20.2%, *n* = 82; practice: 27.4%, *n* = 178). A larger proportion of injuries were diagnosed as contusions in competitions than practices (IPR = 1.89; 95%CI: 1.45–2.46). A larger proportion of injuries were diagnosed as strains in practices than competitions (IPR = 1.35; 95%CI: 1.07–1.70). In addition, 7.4% (*n* = 30) and 4.2% (*n* = 27) of injuries in competitions and practices, respectively, were concussions, with a larger proportion of injuries diagnosed as concussions in competitions than practices (IPR = 1.78; 95%CI: 1.08–2.95).

**Table 4:** Injury counts, percentages, and rates per 1000 Athlete-Exposures (AEs) by diagnosis and type of event in NCAA Men’s Lacrosse, 2009/10–2014/15^a^

	Competition				Practice			
Diagnosis	Injury count (%)	Rate and 95% CI (per 1000AE)	% NTL^b^	% Severe^c^	Injury count (%)	Rate and 95% CI (per 1000AE)	% NTL^b^	% Severe^c^
Concussion	30 (7.4)	0.91 (0.59, 1.24)	10.0	10.0	27 (4.2)	0.16 (0.10, 0.22)	3.7	11.1
Contusion	99 (24.4)	3.02 (2.42, 3.61)	64.6	2.0	84 (12.9)	0.50 (0.40, 0.61)	59.5	2.4
Dislocation	7 (1.7)	0.21 (0.06, 0.37)	42.9	42.9	10 (1.5)	0.06 (0.02, 0.10)	0.0	30.0
Fracture	20 (4.9)	0.61 (0.34, 0.88)	5.0	55.0	24 (3.7)	0.14 (0.09, 0.20)	16.7	62.5
Inflammatory condition	8 (2.0)	0.24 (0.07, 0.41)	50.0	0.0	69 (10.6)	0.41 (0.32, 0.51)	75.4	7.2
Laceration	9 (2.2)	0.27 (0.10, 0.45)	44.4	11.1	7 (1.1)	0.04 (0.01, 0.07)	71.4	0.0
Spasm	9 (2.2)	0.27 (0.10, 0.45)	88.9	0.0	25 (3.8)	0.15 (0.09, 0.21)	76.0	0.0
Sprain	109 (26.9)	3.32 (2.70, 3.95)	43.1	14.7	154 (23.7)	0.93 (0.78, 1.07)	39.0	14.3
Strain	82 (20.2)	2.50 (1.96, 3.04)	43.9	11.0	178 (27.4)	1.07 (0.91, 1.23)	46.1	5.6
Stress fracture	2 (0.5)	0.06 (0.00, 0.15)	0.0	50.0	3 (0.5)	0.02 (0.00, 0.04)	0.0	33.3
Subluxation	14 (3.5)	0.43 (0.20, 0.65)	35.7	0.0	6 (0.9)	0.04 (0.01, 0.06)	50.0	16.7
Other	16 (4.0)	0.49 (0.25, 0.73)	81.3	12.5	63 (9.7)	0.38 (0.29, 0.47)	50.8	7.9
Total	405 (100.0)	12.35 (11.14, 13.55)	46.4	11.9	650 (100.0)	3.90 (3.60, 4.21)	47.4	10.3

NOTE: *NTL* non-time loss

^a^Data originates from the Datalys Center for Sports Injury Research and Prevention Injury Surveillance Program, 2009/10-2014/15

^b^Includes injuries that resulted in participation restriction under 24 h

^c^Includes injuries that resulted in participation restriction of over 3 weeks

In both competition and practice, muscle spasms were the injuries with the largest proportion of NTL injuries (competition: 88.9%, practice: 76.0%). The diagnoses with the largest proportions of severe injuries were fractures (competition: 55.0%, practice: 62.5%).

Concussions comprised the majority of head/face injuries in competition (71.4%, *n* = 30) and practice (64.3%, *n* = 27). The remaining 12 head/face injuries in the competition were six facial lacerations, four contusions, one jaw fracture, and one unspecified head injury. The remaining 15 head/face injuries in practice were four jaw fractures, four facial lacerations, three contusions, one nasal fracture, one jaw subluxation, one headache, and one ruptured eardrum. No eye injuries were reported. In addition, 14 of these 27 non-concussion head/face injuries were NTL.

### Injury mechanism

Contact-related mechanisms comprised large proportions of injuries in competitions (64.2%, *n* = 260) and practices (40.6%, *n* = 264; Table 5). In competitions, the largest proportion of injuries were due to player contact (32.8%, *n* = 133) and non-contact (29.6%, *n* = 120). In practices, non-contact (40.0%, *n* = 260), player contact (17.5%, *n* = 114), and overuse (16.2%, *n* = 105). Contact with the ball and stick comprised 20.7% (*n* = 84) of competition and 14.3% (*n* = 93) of practice injuries.

**Table 5:** Injury counts, percentages, and rates per 1000 Athlete-Exposures (AEs) by injury mechanism and type of event in NCAA Men’s Lacrosse, 2009/10–2014/15^a^

	Competition				Practice			
Injury Mechanism	Injury count (%)	Rate and 95% CI (per 1000AE)	% NTL^b^	% Severe^c^	Injury count (%)	Rate and 95% CI (per 1000AE)	% NTL^b^	% Severe^c^
Player contact	133 (32.8)	4.05 (3.37, 4.74)	40.6	13.5	114 (17.5)	0.68 (0.56, 0.81)	40.4	10.5
Surface contact	40 (9.9)	1.22 (0.84, 1.60)	40.0	12.5	56 (8.6)	0.34 (0.25, 0.42)	39.3	19.6
Ball contact	17 (4.2)	0.52 (0.27, 0.76)	47.1	5.9	47 (7.2)	0.28 (0.20, 0.36)	23.4	10.6
Stick contact	67 (16.5)	2.04 (1.55, 2.53)	58.2	6.0	46 (7.1)	0.28 (0.20, 0.36)	45.7	13.0
Other equipment contact	3 (0.7)	0.09 (0.00, 0.19)	0.0	66.7	1 (0.2)	0.01 (0.00, 0.02)	0.0	100.0
Non-contact	120 (29.6)	3.66 (3.00, 4.31)	46.7	15.0	260 (40.0)	1.56 (1.37, 1.75)	50.0	8.5
Overuse	14 (3.5)	0.43 (0.20, 0.65)	64.3	0.0	105 (16.2)	0.63 (0.51, 0.75)	64.8	7.6
Illness/infection	0	0.00	--	--	7 (1.1)	0.04 (0.01, 0.07)	28.6	14.3
Other/unknown	11 (2.7)	0.34 (0.14, 0.53)	54.5	0.0	14 (2.2)	0.08 (0.04, 0.13)	57.1	7.1
Total	405 (100.0)	12.35 (11.14, 13.55)	46.4	11.9	650 (100.0)	3.90 (3.60, 4.21)	47.4	10.3

NOTE: *NTL* non-time loss

^a^Data originates from the Datalys Center for Sports Injury Research and Prevention Injury Surveillance Program, 2009/10-2014/15

^b^Includes injuries that resulted in participation restriction under 24 h

^c^Includes injuries that resulted in participation restriction of over 3 weeks

Most player contact injuries were sustained to the head/face and shoulder (each 20.6%, *n* = 51) and resulted in sprains (33.2%, *n* = 82), contusions (23.1%, *n* = 57), and concussions (15.8%, *n* = 39). In addition, of the 42 checking-related injuries (4.0% of all injuries), most were sustained to the shoulder (47.6%, *n* = 20) and resulted in sprains (47.6%, *n* = 20). With ball contact injuries, most were sustained to the head/face (31.3%, *n* = 20) and resulted in contusions (46.9%, *n* = 30) and concussions (15.6%, *n* = 10). With stick contact injuries, most were sustained to the hand/wrist (38.9%, *n* = 44) and resulted in contusions (60.2%, *n* = 68). With non-contact injuries, most were sustained to the thigh (24.2%, *n* = 92), ankle (16.8% *n* = 64), and knee (15.3%, *n* = 58), and resulted in strains (47.1%, *n* = 179) and sprains (26.3%, *n* = 100). With overuse injuries, most were sustained to the lower leg (27.7%, *n* = 33) and resulted in inflammatory conditions (37.0%, *n* = 44) and strains (34.5%, *n* = 41).

Compared to practices, competitions had larger proportions of injuries due to player contact (IPR = 1.87; 95%CI: 1.51–2.33) and stick contact (IPR = 2.34; 95%CI: 1.64–3.33). Compared to competitions, practices had larger proportions of injuries due to ball contact (IPR = 1.72; 95%CI: 1.00–2.96), non-contact (IPR = 1.35; 95%CI: 1.13–1.61), and overuse (IPR = 4.67; 95%CI: 2.71–8.05).

In competitions, the injury mechanism with the largest proportion of injuries that were NTL was overuse (competition: 64.3%; practice: 64.8%). The injury mechanisms with the largest proportions of injuries that were severe were non-contact in competition (15.0%) and surface contact in practice (19.6%).

### Common injuries

The most common injury in both competitions and practices were ankle sprains (competition: 12.8%, *n* = 52; practice: 11.2%, *n* = 73; Table 6). Other common injuries included upper leg strains and knee sprains. Knee sprains had the largest proportion of injuries that were severe in both competition (60.9%) and practice (47.2%).

**Table 6:** Common injuries in NCAA Men’s Lacrosse, by event type, 2009/10–2014/15^a^

Injury	n (%)	Rate and 95% CI (per 1000AE)	% NTL^b^	% Severe^c^	Most common injury mechanism, n (% within injury)
Competition					
Ankle sprain	52 (12.8)	1.59 (1.15, 2.02)	50.0%	1.9%	Non-contact, 32 (61.5%)
					Player contact, 11 (21.2%)
Concussion	30 (7.4)	0.91 (0.59, 1.24)	10.0%	10.0%	Player contact, 22 (73.3%)
					Stick contact, 3 (10.0%)
Upper leg strain	29 (7.2)	0.88 (0.56, 1.21)	31.0%	10.3%	Non-contact, 27 (93.1%)
Knee sprain	23 (5.7)	0.70 (0.41, 0.99)	13.0%	60.9%	Player contact, 9 (39.1%)
					Non-contact, 7 (30.4%)
Trunk contusion	19 (4.7)	0.58 (0.32, 0.84)	68.4%	0.0%	Stick contact, 8 (42.1%)
					Player contact, 4 (21.1%)
Practice					
Ankle sprain	73 (11.2)	0.44 (0.34, 0.54)	37.0%	5.5%	Non-contact, 29 (39.7%)
					Player contact, 18 (24.7%)
Upper leg strain	69 (10.6)	0.41 (0.32, 0.51)	31.9%	4.4%	Non-contact, 53 (76.8%)
					Overuse, 14 (20.3%)
Hip/groin strain	50 (7.7)	0.30 (0.22, 0.38)	50.0%	2.0%	Non-contact, 39 (78.0%)
					Overuse, 9 (18.0%)
Knee sprain	36 (5.5)	0.22 (0.15, 0.29)	19.4%	47.2%	Non-contact, 21 (58.3%)
					Player contact, 9 (25.0%)
Lower leg inflammation	32 (4.9)	0.19 (0.13, 0.26)	65.6%	12.5%	Overuse, 23 (71.9%)
					Non-contact, 9 (28.1%)

NOTE: *NTL* non-time loss

^a^Data originates from the Datalys Center for Sports Injury Research and Prevention Injury Surveillance Program, 2009/10-2014/15

^b^Includes injuries that resulted in participation restriction under 24 hours

^c^Includes injuries that resulted in participation restriction of over 3 weeks

## Discussion

This study expands the current literature on collegiate lacrosse injury rates through the use of a more recent injury surveillance data collection platform that incorporated the electronic medical record systems ATs used for their daily clinical practice and eliminated double-data entry (Kerr et al. 2014). These data include NTL injuries, which had not been previously captured in past injury surveillance efforts (Dick et al. 2007a; Xiang et al. 2014). These findings provide a wider breadth of the type of injuries sustained by men’s lacrosse athletes and managed by ATs.

Previous research has examined men’s lacrosse injury data from the NCAA-ISP during a 15-year span (1988/1989–2003/2004), estimating competition and practice injury rates to be 12.58 and 3.24/1000AE, respectively (Dick et al. 2007b). However, such previous research only captured TL injuries, whereas the current study captured both TL and NTL injuries. When considering the TL injuries only, our injury rates (competition and practice injury rates of 6.43 and 2.01/1000AE, respectively) were lower. Our data may highlight a promising trend of decreasing injury rates, based upon two large sets of data separated by a decade. Because of varying samples and data collection strategies, we cannot directly ascertain whether these differences indicate a true decrease in injury rates.

The potential decrease may be associated with equipment and rule changes that aimed to increase athlete safety and health (US Lacrosse 2016a, 2016b). This includes the implementation of preventative exercise programs and changes in equipment, such as lighter helmets and titanium alloys being used in the shafts of lacrosse sticks to reduce their mass and make them less prone to breakage. US Lacrosse also provides position statements on helmets, mouth guards, and sticks, as well as guidelines for equipment fit (US Lacrosse 2017). With the continued increase of participation at youth, high school, and collegiate levels (National Collegiate Athletic Association 2015; National Federation of High Schools 2015; US Lacrosse 2016b), more advanced skilled players may be reaching the collegiate level and may have better movement patterns, awareness of body positioning, and control of the stick. With athletes starting participation at a younger level, they may enter the collegiate level of play better trained and less at risk for injury. However, it is possible that unintended adverse effects may be produced from early specialization (Feeley et al. 2016) as well as rule changes (Westermann et al. 2016). Because the NCAA-ISP did not specifically collect information regarding their implementation of such programming, use of such equipment, or playing histories, we cannot ascertain a direct association between these and changes in injury incidence. Nevertheless, our findings are promising and may indicate the potential for success of injury prevention strategies in men’s lacrosse.

Previous research has found that competition injury rates were higher than practice injury rates (Dick et al. 2007a; Hinton et al. 2005; McCulloch and Bach 2007; Xiang et al. 2014). Even with a more inclusive injury definition (including NTL injuries), our study found a similar competition vs. practice injury rate ratio. As practices are typically controlled environments in which skill development and preparation for competitions occur, the intensity of effort is typically lower than that of competitions, which may explain the reduced injury rates. However, the overall count of injuries was higher in practices than competitions, mostly due to the increased exposure time in practices. Because practices are settings in which coaching staff can direct and correct athlete behavior, they can serve as venues to implement potentially beneficial injury prevention interventions (Kerr et al. 2015). Thus, it is as important to consider injury prevention within all settings of men’s lacrosse to have the greatest impact in injury incidence reduction.

Preseason practices continue to have a larger injury rate than regular season practices (Agel and Schisel 2013; Dick et al. 2007b; Hootman et al. 2007). Previous research has hypothesized numerous reasons for this disparity, including athletes coming into the preseason poorly conditioned or as “walk-ons” with less skill development, the increased likelihood of longer sessions or multiple sessions per day in the preseason versus the regular season, and more competitiveness among athletes as they compete for roster spots or starting positions (Agel and Schisel 2013; Hootman et al. 2007). From a surveillance standpoint, Hootman et al. (2007) advocated for more in-depth examinations of the “intensity” of exposure related to practices; Agel and Schisel (2013) highlight that although there is an awareness in this difference between preseason and regular season injury rates, future research needs to explore the factors that drive this disparity. We support the previous calls and advocate for future research efforts that examine the type of activities occurring within practice sessions that may place athletes at increased injury risk. Because lacrosse is a spring sport, the preseason occurs in the winter months, in which weather may create poor field conditions. However, higher preseason practice injury rates are not exclusive to men’s lacrosse and have been seen in other sports (Hootman et al. 2007; Kerr et al. 2016a). Thus, the NCAA may benefit from examining safety-related rule changes that may reduce injury incidence in preseason practices; a standard could originate from recent guidelines from football that recommended limiting the amount of contact that occurs during preseason practices (National Collegiate Athletic Association 2014b).

Most men’s lacrosse injuries occurred to the lower extremity, which is similar to previous research (Dick et al. 2007a). Lacrosse is a lower extremity-intensive sport in which student-athletes are actively sprinting, cutting, starting and stopping, and pivoting. Throughout participation, there is the risk for potential contact with other players and equipment, as indicated by our findings that 64.2% of competition and 40.6% of practice injuries were due to contact (with players, the surface, and equipment). Although men’s lacrosse student-athletes are equipped with padded gloves, elbow and chest/shoulder pads, and helmets, the lower extremity has minimal protection from contact-related injuries.

As seen in previous research (Dick et al. 2007a; Vincent et al. 2015), ankle sprains continue to be the most common injury in both competitions (12.8%) and practices (11.2%). The findings highlight the need for prevention strategies to mitigate the incidence of such injuries in men’s lacrosse. Traditional ankle taping or semi rigid bracing of the ankle may decrease the rate of persistent ankle sprains (Kaminski et al. 2013). In addition, evaluating and strengthening the lower leg muscles that invert, evert, dorsiflex, and plantarflex the ankle as well as hip abductors and extensors may prevent ankle injury (Kaminski et al. 2013). Lastly, it has been found that limited dorsiflexion range of motion can lead to higher rates of ankle injury (Kaminski et al. 2013). Sports medicine clinicians should consider the use of such evaluation and prevention strategies to mitigate athlete risk of ankle sprains in lacrosse.

Knee sprains were prevalent in men’s lacrosse and comprised the majority of severe injuries. Non-contact mechanisms were the most common in cases of knee ligament sprains and were commonly listed as the mechanism in TL injuries. There are several ligamentous structures within the knee, such as the anterior cruciate ligament (ACL) and medial collateral ligament (MCL), which can be injured in this type of mechanism, singularly or combined. Sprains to these ligaments can be severe due to the significant amount of time to heal or required surgical intervention (Agel et al. 2016; Stanley et al. 2016; Swenson et al. 2013). Although ACL injury rates, as well as general knee injury rates, have been found to be higher in females and males (Agel et al. 2016; Stanley et al. 2016; Swenson et al. 2013), it is essential to develop, implement, and evaluate knee injury prevention strategies. Knee sprains have been shown to decrease with the use of preventative exercise programs such as a warm-up involving slow, controlled cuts, core strengthening, and hip mobility (Grimm et al. 2015; US Lacrosse 2016b). By teaching proper landing and take-off techniques, injury rates can be decreased, especially non-contact injuries. Programs that include dynamic warm-ups and emphasize core strength, balance, and proper landing techniques, are available (US Lacrosse 2016b).

As in previous research (Dick et al. 2007a; Lincoln et al. 2007; Marshall et al. 2015), there were a number of head/face injuries sustained by men’s lacrosse athletes, with the majority being concussions. However, other head/face injuries were reported, including fractures, lacerations, and contusions. Concussion rates (0.86 and 0.15/1000AE in competitions and practices, respectively) were not as high as in other sports reported in the NCAA-ISP, such as football, ice hockey, and soccer (Zuckerman et al. 2015). Stil, the findings demonstrate the need for concussion prevention and awareness to ensure appropriate identification, diagnosis, management, and return to play. Rule and equipment changes may also be needed, given that many player and stick contact injuries were concussions. The NCAA added a prohibition of players targeting the head and neck for the 2011 season, and asked referees to more stringently penalize head and neck contact (National Collegiate Athletic Association 2010), There have been no additional rule changes or rules emphasis directly pertaining to concussions, body checks or hits the head since then. However, a rule regarding head/neck targeting has had the term “deliberate” removed from the infraction definition that went into effect during the summer of 2016 (National Collegiate Athletic Association 2014a), This rule change means that any athlete who has intentional/unintentional contact to an opponent’s head can be penalized and ultimately ejected. The lacrosse ball also has contact with players’ helmets, which is difficult to avoid. The National Operating Committee on Standards for Athletic Equipment (NOCSAE) has recently modified the lacrosse ball compression standard (NOCSAE 2016), which would decrease the force/impact on the head and body, while not affecting gameplay. Concussion rates should be re-evaluated after these rule and equipment changes have been implemented for an appropriate amount of time.

Many player contact injuries and checking-related injuries were sustained to the upper extremity (e.g., shoulder, elbow, arm/wrist) and were diagnosed as contusions and sprains. Previous research has posited this association between contact and upper extremity injuries (Bowers et al. 2010; Gardner et al. 2016; Vincent et al. 2015). Although the incidence is lower than estimated in previous research from the 2004/05–2008/09 academic years (Gardner et al. 2016), the findings highlight the need to explore injury prevention approaches to protect the shoulder. Body checking is permitted if the opponent has the ball or is within five yards of a loose ball. All body contact must occur from the front or side, above the waist and below the shoulders. In 2015, a new rule was integrated making it illegal to use a body part (upper or lower) to initiate contact with an opponent’s stick or his own stick (National Collegiate Athletic Association 2014c). Ensuring and reinforcing proper checking skills may help to mitigate injury risk. Despite our findings, we were unable to differentiate the types of player contact and checking that may have been associated with injury. Future research should aim to examine whether player contact was done legally or illegally and whether it involved body checking (i.e., hitting or pushing another player with any part of one’s body), cross checking (i.e., using the portion of the stick shaft, between one’s hands, to hit or push another player), or stick checking (i.e., using the head or shaft of stick to hit another player or their stick). Such detail will help to identify specific areas of gameplay where injury prevention can be focused.

From a surveillance perspective, there is a need for additional recruitment of teams to contribute injury data to provide sufficient statistical power to analyze the effects of these rule changes. Currently, the sample size may be insufficient to detect differences in injuries with lower counts, such as concussions and other head/face injuries. As had been done in other sports (Clifton et al. 2016; Dompier et al. 2015; Kerr et al. 2016b), future studies should implement similar data collection methodologies to compare risk and mechanisms of injury among male lacrosse players at the youth, high school, and professional levels of play.

### Limitations

The NCAA-ISP relies upon a convenience sample of all men’s lacrosse programs. As a result, these data may not be generalizable to non-participating programs. Our findings may also not be generalizable to lacrosse within other levels of competition (e.g., high school, professional, junior colleges). Other limitations include the exclusion of injuries that occurred outside of school-sanctioned practices and competitions and during the off-season, and of the type of care that injured student-athletes received at the time of injury. An athlete-exposure was unit-based, and we were unable to interpret injury rates per minute or hour of participation. However, the use of AEs was done to reduce burden of data collection on the ATs.

## Conclusions

The TL injury rates in NCAA men’s lacrosse reported in the present study are lower than those previously reported (Dick et al. 2007b). Such a decrease may be associated with rule changes and injury prevention programming such as preventative exercise programs and ankle bracing/taping/strengthening. Continued development and implementation of injury prevention programming is necessary to further reduce the risk of injury, particularly related to lower extremity injuries and concussions, which contribute the largest proportions of injuries.

As lacrosse grows in popularity, more research is essential to protecting the health and safety of lacrosse players (McCulloch and Bach 2007). Future data collection may benefit from including additional variables that examine specific injury prevention components such as individual player usage of protective equipment and team implementation of training interventions. Surveillance also could consider additional aspects that may be related to increased injury risk, including the type of checking (i.e., body vs. cross vs. stick) causing injury and specific activities per practice session. Although the feasibility of collecting such data must be examined prior to implementation, such additions may help to better understand the effects of such prevention interventions on injury incidence.

## Abbreviations

AE: Athlete-exposure
AT: Athletic trainer
IPR: Injury proportion ratio
IRR: Injury rate ratio
NCAA: National Collegiate Athletic Association
NCAA-ISP: National Collegiate Athletic Association Injury Surveillance Program
NTL: Non-time loss
TL: Time loss
